# The Histone Chaperone HIRA Is a Positive Regulator of Seed Germination

**DOI:** 10.3390/ijms22084031

**Published:** 2021-04-14

**Authors:** Elodie Layat, Marie Bourcy, Sylviane Cotterell, Julia Zdzieszyńska, Sophie Desset, Céline Duc, Christophe Tatout, Christophe Bailly, Aline V. Probst

**Affiliations:** 1 IBPS, UMR 7622 Biologie du Développement, CNRS, Sorbonne Université, 75005 Paris, France; elodie.layat@gmail.com (E.L.); marie_bourcy@hotmail.com (M.B.); christophe.bailly@upmc.fr (C.B.); 2 iGReD, CNRS, Inserm, Université Clermont Auvergne, 63000 Clermont-Ferrand, France; sylviane.cotterell@uca.fr (S.C.); sophie.desset@uca.fr (S.D.); christophe.tatout@uca.fr (C.T.); 3 Department of Plant Physiology, Institute of Biology, Warsaw University of Life Sciences–SGGW, 02-776 Warsaw, Poland; julia.zdzieszynska@gmail.com; 4 UFIP UMR-CNRS 6286, Épigénétique et Dynamique de la Chromatine, Université de Nantes, 2 rue de la Houssinière, 44322 Nantes, France; Celine.Duc@univ-nantes.fr

**Keywords:** seed dormancy and germination, chromatin, histone variants, histone chaperones, seed vigor

## Abstract

Histone chaperones regulate the flow and dynamics of histone variants and ensure their assembly into nucleosomal structures, thereby contributing to the repertoire of histone variants in specialized cells or tissues. To date, not much is known on the distribution of histone variants and their modifications in the dry seed embryo. Here, we bring evidence that genes encoding the replacement histone variant H3.3 are expressed in Arabidopsis dry seeds and that embryo chromatin is characterized by a low H3.1/H3.3 ratio. Loss of HISTONE REGULATOR A (HIRA), a histone chaperone responsible for H3.3 deposition, reduces cellular H3 levels and increases chromatin accessibility in dry seeds. These molecular differences are accompanied by increased seed dormancy in *hira-1* mutant seeds. The loss of HIRA negatively affects seed germination even in the absence of HISTONE MONOUBIQUITINATION 1 or TRANSCRIPTION ELONGATION FACTOR II S, known to be required for seed dormancy. Finally, *hira-1* mutant seeds show lower germination efficiency when aged under controlled deterioration conditions or when facing unfavorable environmental conditions such as high salinity. Altogether, our results reveal a dependency of dry seed chromatin organization on the replication-independent histone deposition pathway and show that HIRA contributes to modulating seed dormancy and vigor.

## 1. Introduction

Seeds are central to plant reproduction and the dispersal of plant species, and critical for human nutrition, accounting for approximately 70% of energy intake of the human population. The seed is a particular developmental stage with low moisture and devoid of metabolic activities including transcription and *de novo* protein synthesis [1]; this quiescent stage allows survival under unfavorable conditions such as high and low temperatures. Embryo development, triggered by fertilization of egg and central cell, is completed before the seed enters maturation and finally transits to the desiccation phase. The seed’s ability to germinate in a wide range of agroclimatic conditions, usually termed as seed vigor, is crucial for seedling emergence and establishment in crop species [2]. Seed vigor results from a complex interaction of seed properties that are affected by genetics, conditions of seed development on the mother plant, seed harvesting processes and conditions of storage after harvest [3]. In addition, an intrinsic biological process, termed seed dormancy, may delay seed germination even when favorable environmental conditions are met [1]. Dormancy controls the precise timing of germination to ensure post-germination development of the emerging seedling in appropriate conditions; therefore, diverse environmental signals are integrated to decide when to initiate seed germination [4]. Seed dormancy is mostly controlled by the antagonistic relationship between the plant hormones abscisic acid (ABA), which promotes dormancy, and gibberellins (GAs), which promote germination [5,6]. Many molecular regulators of seed dormancy have also been identified so far. They include actors of ABA and GAs metabolism and signaling pathways [6] but also specific genes such as *DELAY OF GERMINATION 1 (DOG1)*, a quantitative trait locus controlling seed dormancy [7]. 

Exemplified in the model plant *Arabidopsis thaliana* [8], nuclear and chromatin organization undergo drastic changes during seed development and germination, with dry seeds being characterized by particularly small nuclei with highly condensed chromatin. Starting with the uptake of water during imbibition, the dry and quiescent seed will reactivate metabolism, correlated with an increase in nuclear size and the progressive decondensation of chromatin [8,9]. At the molecular level, germination requires a general re-starting of previously paused protein translation, associated with the degradation of stored mRNAs accumulated during seed maturation [10,11] and down-regulation of dormancy related genes [12]. In the context of the seed, a measured balance between packaging of the large linear DNA molecules into chromatin, which allows protection of the genetic material during seed desiccation, and the control of transcription during maturation is thought to regulate expression of genes involved in dormancy establishment [13]. Seed development is therefore expected to involve chromatin reorganization from nuclear architecture down to changes in the composition and modifications of the basic subunit of chromatin, the nucleosome. The nucleosome allows the first degree of chromatin packaging as ~147 bp of DNA wrap around an octamer of the core histones H2A, H2B, H3 and H4 [14]. Any structural alterations to the nucleosome, such as covalent modifications of histone proteins or the incorporation of different histone variants, can affect the strength of association of histones with DNA and modulate DNA accessibility and higher-order chromatin organization [15,16]. Furthermore, combinations of histone post-translational modifications (PTMs), DNA methylation and histone variants contribute to the formation of different chromatin states [17,18]. Since transcription requires access to the DNA template and nucleosome disassembly, which can be facilitated or inhibited by different chromatin states, these combinations of epigenetic marks influence local transcriptional outcomes. Chromatin organization is therefore anticipated to contribute to the establishment and maintenance of the dormancy state of the seed. Indeed, mutants lacking several proteins involved in the setting of histone modifications show altered dormancy phenotypes. For example, seeds lacking the H3K9 histone methyltransferases KRYPTONITE (SUVH4/KYP) show increased seed dormancy [19]. Histone deacetylases also contribute to seed dormancy regulation, with HDA9 being a negative regulator of germination [20] while HDA6 and HDA19 contribute to the repression of embryogenic properties and therefore promote germination [21]. Furthermore, mutants in HISTONE MONOUBIQUITINATION 1 (HUB1), an E3 ligase involved in histone H2B monoubiquitination, a histone mark associated with transcriptional activity, show reduced seed dormancy [22]. Both histone acetylation and monoubiquitination have the intrinsic capacity to modify the chromatin structure. They interfere with nucleosome-array folding and facilitate DNA accessibility by loosening histone-DNA charge contacts or by modifying the nucleosome structure, respectively [23]. Histone H2B monoubiquitination facilitates transcription elongation and eases nucleosome reassembly [24,25] and its loss leads to lower *DOG1* transcript levels in seeds. Altered *DOG1* expression was also observed for seeds lacking the TRANSCRIPTION ELONGATION FACTOR II S (TFIIS) / REDUCED DORMANCY 2 (RDO2) [26,27], a component of the RNA Polymerase II transcript elongation complex [28]. 

At the level of the nucleosome, the co-occurrence of specific histone variants such as the H3 histone variant H3.3 and the H2A variant H2A.Z can result in unstable nucleosomes [29], and in Arabidopsis, H3.3 occupancy is correlated with RNA pol II association and elevated transcriptional activity [30,31,32]. The correct incorporation of histone variants in time and space depends on evolutionary highly conserved histone chaperone complexes, some of these protein complexes being able to selectively transport or deposit different core histones and even specific histone variants [16,33]. In contrast to the replicative variant H3.1, which is assembled into chromatin in a DNA-synthesis-dependent manner, the replacement variant H3.3 is deposited in a DNA-synthesis-independent manner [34]. The CHROMATIN ASSEMBLY FACTOR 1 (CAF-1) complex [35,36] is responsible for H3.1 deposition at the replication fork [37,38] and DNA repair sites [39]. In contrast, H3.3 is deposited through DAXX-ATRX and the HISTONE REGULATOR A (HIRA) chaperone [40,41], which functions in a complex together with UBN and CABIN [42,43]. Mammalian HIRA ensures both *de novo* deposition and recycling of histone H3.3 [44]. In plants, H3.3 deposition also depends on HIRA [42,43] and ATRX [32] with the HIRA-dependent pathway playing a major role. 

While in heterogeneous seedling tissue both H3.1 and H3.3 co-exist, with H3.1 being enriched in heterochromatin and H3.3 at active genes and regulatory regions [30,31,45], certain cell types show preferential enrichment in either H3.1 or H3.3 or in other atypical H3 variants [46,47]. For example, in root tissues, cells that undergo their last cell division before cell proliferation arrest evict H3.1 and incorporate H3.3 [48]. The absence of *H3.1* transcripts and protein have also been observed in mature pollen and no H3.1 protein was found in the egg cell, which expresses high levels of H3.3 [46], together revealing various dynamics of the H3.1/H3.3 balance in different plant tissues. 

Here, we show that mostly replacement histone H3 variants and their deposition machinery are expressed in dry seeds and that embryonic nuclei are enriched in the replacement variant H3.3. Loss of HIRA leads to reduced H3 histone content and renders dry seed chromatin more accessible to MNase digestion. While *hira-1* mutant seeds are viable under standard growth conditions, they show increased dormancy, reduced viability when artificially aged and impaired germination under high salinity, identifying HIRA as a positive regulator of germination.

## 2. Results

### 2.1. Dry Seed Chromatin Is Enriched in the Replacement Variant H3.3

After fertilization, the zygote undergoes cell divisions, forming the embryo before cell proliferation ceases and the embryo matures, building the dry seed. Since the incorporation and exchange of histone variants have been associated with developmental transitions [46,47] or with cells with different proliferation potential [48], we were interested to investigate whether similar changes in the histone H3 variant repertoire would occur during seed maturation, imbibition and germination. Five genes (*HTR1, 2, 3, 9* and *13*) encode H3.1, while three genes (*HTR4, 5* and *8*) encode H3.3. We quantified the transcript levels of these genes in flowers, siliques at different developmental stages of seed formation, dry seeds and seeds imbibed from 2 to 32 h as well as seedlings two days after germination.

**Figure 1:**
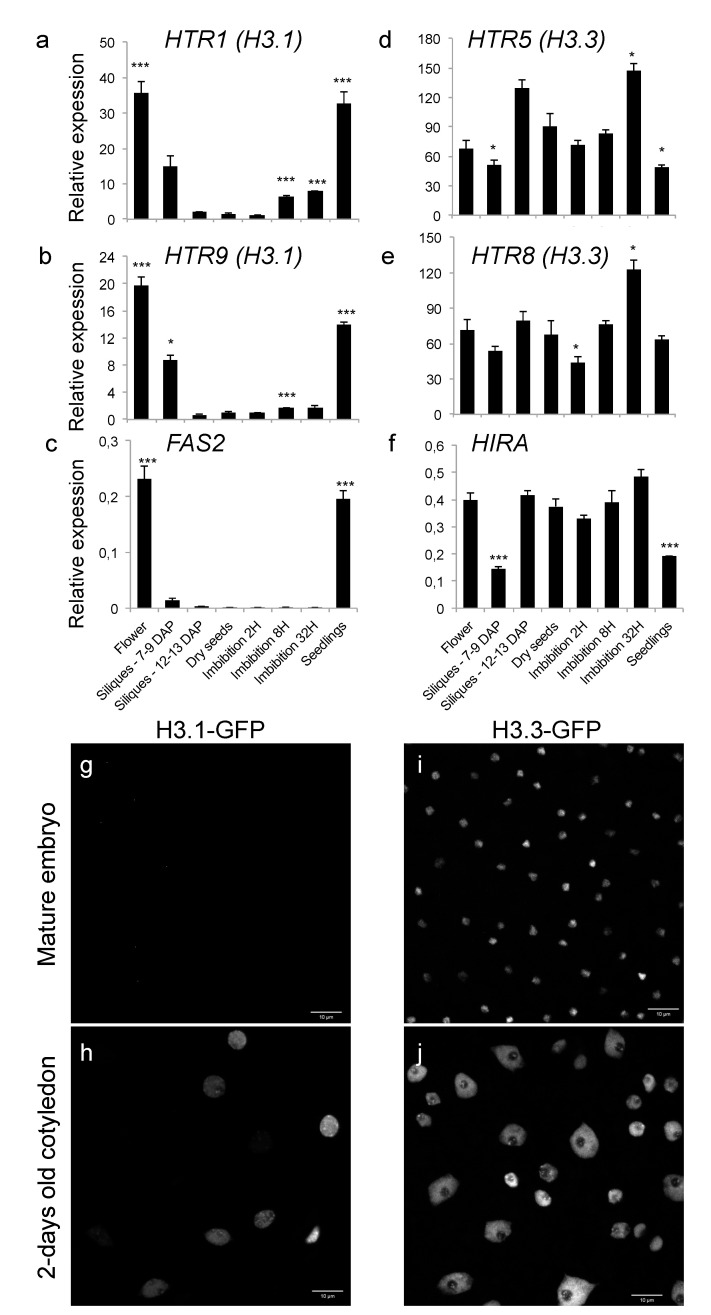
Chromatin in dry seeds is enriched in the replacement histone H3.3 variant. (**a**–**f**) Relative transcript levels of two genes encoding H3.1 (*HTR1* (**a**), *HTR9* (**b**)) and the middle subunit of the CAF-1 complex (*FAS2* (**c**)), as well as two genes encoding H3.3 (*HTR5* (**d**), *HTR8* (**e**)) and the central subunit of the HIR complex (*HIRA* (**f**)) in flowers, siliques (7 to 9 and 12 to 13 days after pollination (DAP)), dry seeds and seeds imbibed for 2 hours (H), 8 h or 32 h as well as two-days-old seedlings. Transcript levels are normalized to *MONENSIN SENSITIVITY 1* (*MON1*, *At2g28390*). Error bars correspond to the standard error of the mean (SEM) of three biological replicates and statistically significant differences relative to dry seeds were determined using a two-sided Student’s *t*-test (* *p* < 0.05; *** *p* < 0.001). (**g**–**j**) Maximum intensity projections of confocal images of embryos 1 h after imbibition (**g**,**i**) and two-days-old cotyledons (**h**,**j**) of transgenic lines expressing H3.1-GFP (**g**,**h**) or H3.3-GFP (**i**,**j**).

While we found *HTR3* transcript levels to be low at these developmental stages, *HTR1, HTR2, HTR9* and *HTR13* showed a dynamic expression pattern with high transcript levels in flowers, which strongly decreased during seed maturation and were nearly absent in dry seeds before increasing again modestly 8 h after imbibition (Figure 1a,b, Figure S1a–c). This expression pattern is similar to that of the *FASCIATA 2 (FAS2)* gene, encoding the middle subunit of the CAF-1 complex, which is responsible for H3.1 incorporation [9,38]. *FAS2* transcripts are undetectable in late silique stages and only present again in germinated seedlings (Figure 1c). 

In contrast, *HTR4, HTR5* and *HTR8* (encoding H3.3) as well as the gene encoding the central subunit of the HIR complex (*HIRA*) are expressed throughout these different developmental stages (Figure 1d–f and Figure S1d) indicating that the H3.3 incorporation pathway is active in maturing and dry seedlings. These expression data are in agreement with published RNA-seq data of dry and imbibed seeds [49] illustrating that the DNA replication-dependent histone deposition machinery as well as most of the H3.1 encoding genes are not expressed in dry seeds (Figure S2a,b), while transcripts of the genes encoding H3.3 or the HIR complex subunits are present at this developmental stage (Figure S2c,d). To confirm these observations at the protein level, we used transgenic plants expressing either H3.1-GFP [50] or H3.3-GFP [46] under control of their endogenous promoters and acquired confocal images of cotyledon nuclei from plantlets two days after germination (dag) and embryos dissected manually from shortly imbibed seeds (Figure 1g–j). While H3.3 is enriched in most nuclei in cotyledons at 2 dag, *HTR1 (H3.1)* is enriched in a subset of nuclei in agreement with previous observations [46,48] (Figure 1h,j). No H3.1-GFP-positive nuclei could be detected in embryonic cotyledons while chromatin of the small and highly condensed nuclei of the embryonic cotyledon was enriched in H3.3 (Figure 1g,i). Thus, chromatin in dry seeds shows a very low H3.1/H3.3 balance, implying that chromatin dynamics during seed maturation and imbibition rely mostly on the H3.3 incorporation pathway.

### 2.2. HIRA Mutant Seeds Show Reduced Histone Content and MNase Hypersensitivity 

**Figure 2:**
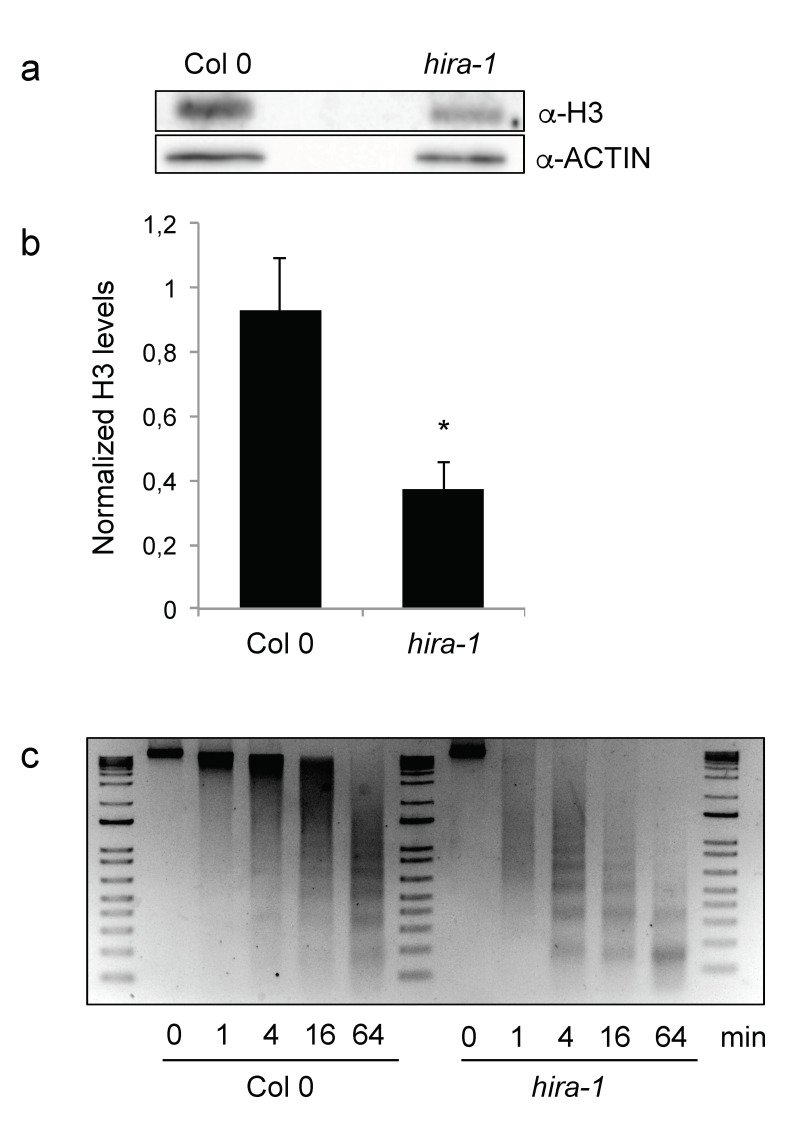
Loss of HIRA leads to reduced H3 content and MNase hypersensitivity. (**a**) Representative Western blot of H3 histones in total protein extracts from WT and *hira-1* mutant dry seeds. (**b**) Quantification of H3 band intensities relative to ACTIN from three biological and four technical replicates. One wild type sample was set to 1 in each technical replicate. Statistically significant differences relative to WT were determined using a two-sided Student’s *t*-test (* *p* < 0.05). (**c**) Nuclei from WT or *hira-1* mutant seeds were isolated and incubated with MNase for 1, 4, 16 and 64 min. Equal amounts of digested DNA were loaded on an agarose gel and stained with ethidium bromide. One experiment of three biological replicates with similar results is shown.

The HIRA chaperone complex is the predominant histone chaperone responsible for DNA-synthesis-independent histone deposition in Arabidopsis [32,43]. Nevertheless, *hira* mutant plants (*hira-1*) show few developmental defects and are fertile [42,43,46]. We could therefore test whether histone levels are affected in *hira-1* mutant seeds, a tissue in which only *H3.3* gene transcripts are present. We first quantified the transcript levels of genes encoding H3.1 (*HTR1, HTR9*) and H3.3 (*HTR5, HTR8*) in *hira-1* mutant seeds versus wild type (WT), revealing higher transcript levels of these four histone genes in the mutant background (Figure S3). Despite elevated H3 transcript levels, H3 protein levels in total protein extracts from *hira-1* mutant seedlings were lower compared to those in WT seeds (Figure 2a,b), therefore strongly suggesting that in absence of the HIRA-mediated H3 deposition and recycling, seed chromatin shows globally reduced nucleosomal occupancy. 

We then asked whether loss of HIRA would lead to altered chromatin organization in seeds. We therefore incubated nuclei isolated from WT and *hira-1* mutant dry seeds with micrococcal nuclease (MNase) for different durations. MNase cleaves linker DNA between nucleosome core particles yielding a sequence of DNA fragments down to mono-nucleosomes. Compared to WT, chromatin from *hira-1* mutant seeds was digested more rapidly by MNase revealing increased global chromatin sensitivity to MNase digestion. Together with the reduced histone H3 content, this suggests that, in the absence of HIRA, H3.3 deposition and recycling cannot be completely ensured by alternative pathways. It further suggests the existence of a different chromatin structure in *hira-1* mutant seeds with reduced nucleosomal occupancy and increased chromatin accessibility.

### 2.3. HIRA Loss of Function Results in Dormancy Defects

**Figure 3:**
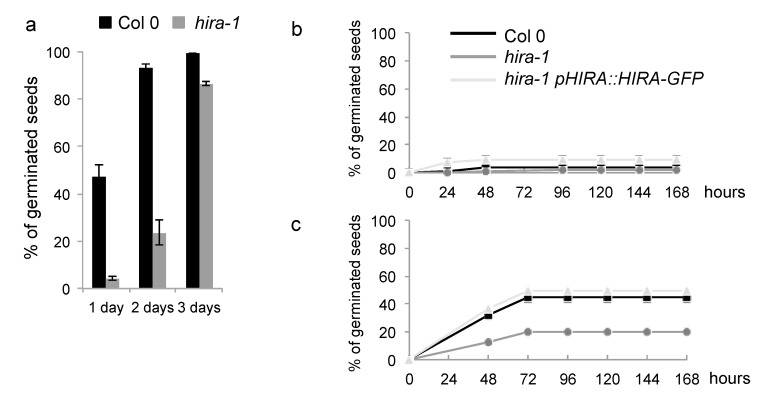
Seeds lacking HIRA show enhanced seed dormancy. Dormancy defects induced by HIRA loss-of-function (**a**) Germination of freshly harvested seeds of Col-0 (black) and *hira-1* mutants (dark grey) in darkness at 25 °C after one, two and three days of stratification. Percentage of germinating seeds was scored six days after transfer to 25 °C. (**b**,**c**) Germination of freshly harvested seeds of Col-0 (black), *hira-1* mutants (dark grey) and the complementing line (*hira-1 pHIRA::HIRA-GFP*; light grey) in darkness at 25 °C without (**b**) and after three days of stratification at 4 °C (**c**). Error bars correspond to SD from three biological replicates.

To reveal the possible functional consequences of altered histone content in *hira-1* mutant seeds, we evaluated germination efficiency of these seeds after different stratification times. When placed at 25 °C in darkness for germination, longer stratification times were necessary to achieve full germination efficiency in *hira-1* mutant seeds compared to WT, revealing increased seed dormancy in *hira-1* mutant seeds (Figure 3a). To confirm these results and to ensure that this phenotype is caused by the loss of HIRA, we tested seed dormancy in independent seed batches of WT and *hira-1* mutant seeds and included seeds from a complementing line expressing HIRA-GFP under control of its endogenous promoter in the *hira-1* mutant background [42]. Freshly harvested seeds of all three genotypes were dormant, as only 5 to 10% of the seeds germinated at 25 °C in darkness (Figure 3b). Stratification at 4 °C for three days increased germination percentages at 25 °C in darkness of WT seeds and seeds from the complementing line to 45% and 50%, respectively, while only 20% of *hira-1* mutant seeds germinated (Figure 3c). Therefore, the loss of HIRA causes stronger seed dormancy (Figure 3). 

### 2.4. Dormancy Defects in hira-1 Mutants Are Unaffected by Plant Hormones 

**Figure 4:**
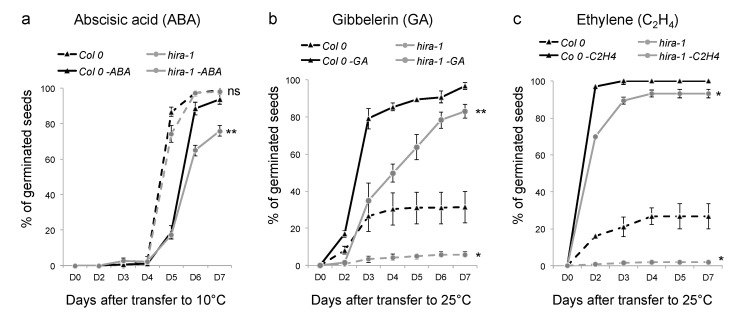
Enhanced seed dormancy of *hira-1* mutants is not alleviated upon treatment with ABA, GA or ethylene. Germination of Col-0 (black, square) and *hira-1* mutant (dark grey, circle) seeds in presence (filled line) or absence (dashed line) of ABA (1 µM) (**a**) at 10 °C in darkness or of GA (1 mM) (**b**) and ethylene (50 ppm) (**c**) at 25 °C in darkness. Means of biological triplicates with SEM are shown. Statistically significant differences relative to WT at D7 were determined using a two-sided Student’s *t*-test (* *p* < 0.05; ** *p* < 0.01; ns = not significant).

As dormancy is controlled by plant hormones, with Abscisic acid (ABA) known to promote dormancy, while gibberellin (GA) and ethylene stimulate germination [51], we tested to what extent the increased dormancy phenotype in *hira-1* mutants is affected by different plant hormones. When placed at 10 °C, 100% of WT and *hira-1* mutant seeds germinated, confirming seed viability. When grown under the same condition in the presence of ABA, the germination rate of both WT and *hira-1* mutant seeds was negatively affected, with a stronger impact on mutant seeds (Figure 4a). 

At 25 °C in the dark, gibberellin and ethylene positively affected the germination of both genotypes, resulting in 100% germination of the WT batches. While GA and ethylene stimulated the germination of *hira-1* mutant seeds, their germination efficiency was lower compared to the WT and only 80% or 90% of seeds germinated in the presence of GA and ethylene, respectively (Figure 4b,c), suggesting that the increased dormancy phenotype in *hira-1* mutants might be related to lower sensitivity to these dormancy-alleviating hormones.

To complement these analyses, we determined transcript levels of several genes involved in ABA, GA and ethylene metabolism in dormant or non-dormant WT and *hira-1* mutant seeds before and after incubation for 16 h or 24 h under light (Figure S4). No significant differences were observed for genes encoding proteins involved in ABA signaling (*ABI5*) or catabolism (*CYP707A),* GA activation (*GA3ox1*) or catabolism (*GA2ox2*) and ethylene signal transduction (*EIN2*), suggesting that *hira-1* seed dormancy defects are not caused by the deregulation of expression of these genes.

### 2.5. HIRA Counteracts the Positive Regulators of Dormancy TFIIS and HUB1 

**Figure 5:**
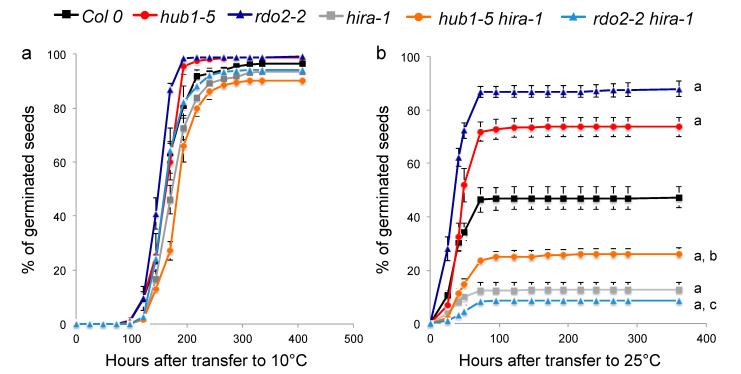
*HIRA* is epistatic to *HUB1* and *RDO2/TFIIS*. (**a**,**b**) Germination of freshly harvested seeds of Col 0 (black), *hub1-5* (red), *rdo2-2* (dark blue), *hira-1* (grey), *hub1-5 hira-1* (orange) and *rdo2-2 hira-1* (light blue) at 10 °C (seed viability test; **a**) or at 25 °C (dormancy test; **b**) in darkness. Means of biological triplicates with SEM are shown. Statistically significant differences relative to dry seeds were determined using a two-sided Student’s *t*-test (a = *p* < 0.01 relative to Col 0; b = *p* < 0.01 relative to *hub1-5*; c = *p* < 0.01 relative to *rdo2-2*).

Altered histone content in the *hira-1* mutant seeds might directly affect gene expression regulation in the dry seed and, consequently, dormancy control. Indeed, a lack of proteins involved in transcription regulation such as RDO2/TFIIS, which is part of the Arabidopsis RNAPII Elongation Complex [28] or RDO4/HUB1, an E3 protein ligase involved in histone H2B monoubiquitination [52], show reduced seed dormancy [22,26,27]. Both have been suggested to facilitate gene expression during late seed maturation stages [53]. To investigate the epistatic relationship between HIRA, TFIIS and HUB1, which all have the potential to affect transcription during seed maturation and germination, we crossed *hira-1* mutants to both *rdo2-2* and *hub1-5* mutants. All double mutant plants were viable and fertile (Figure S5a) and 90 to 100% of their seeds germinated at 10 °C (Figure 5a and Figure S5b). 

When placed at 25 °C in darkness, *rdo2-2* and *hub1-5* seeds show lower and *hira-1* mutant seeds higher dormancy levels than WT seeds, as expected (Figure 5b, Figure S5c). Seeds from the double mutants—*hub1-5 hira-1* and *rdo2-2 hira-1*—are more dormant compared to WT. This shows that *HIRA* is epistatic to *RDO2*/*TFIIS* and *HUB1* as its absence suppresses the negative effect of *rdo2* and *hub1* mutants on seed dormancy. 

### 2.6. Loss of HIRA Reduces Seed Vigor

**Figure 6:**
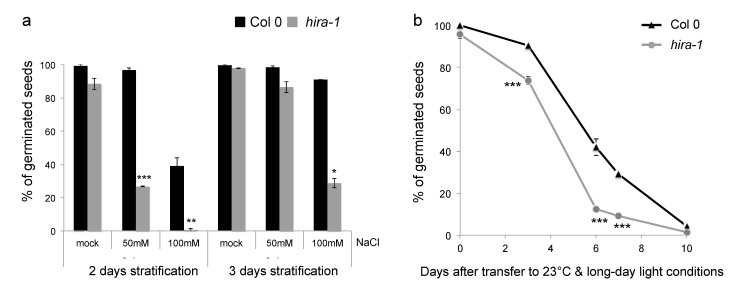
Germination of *hira-1* mutant seeds is affected by salt stress and is sensitive to storage in a high temperature and humidity. (**a**) Germination of Col 0 (black) and *hira-1* (grey) seeds under long-day conditions at 23 °C in presence of 50 mM or 100 mM NaCl after two or three days of stratification at 4 °C in darkness. Means of two biological replicates with SEM are shown. (**b**) Germination of Col 0 (black) and *hira-1* (grey) seeds under long-day condition at 23 °C after exposure of dry seeds for 4, 6, 7 or 10 days to 80% relative humidity at 37 °C. Seeds were stratified for three days at 4 °C in darkness and germinated seeds scored six days after transfer to light. Means of four biological replicates with SEM are shown. Statistically significant differences relative to wild type seeds were determined using a two-sided Student’s *t*-test (* *p* < 0.05, ** *p* < 0.01, *** *p* < 0.001).

Given the reduced histone content and increased MNase sensitivity in *hira-1* mutants, we wanted to explore whether these seeds show reduced vigor when germinated in stressful conditions. We therefore first tested germination in the presence of increased salinity, a stress condition known to strongly affect germination [54]. We observed that germination was affected in a dose-dependent manner in the presence of salt in the growth medium. Seeds lacking HIRA germinated less well when exposed to salt. Indeed, 70% of *hira-1* mutant seeds failed to germinate in the presence of 100 mM salt while over 90% of the WT seeds germinated under these conditions (Figure 6a).

Then, we tested seed longevity, which is known to rapidly decrease under accelerated ageing treatment (storage under high relative humidity and high temperature). To this aim, we incubated WT and *hira-1* mutant seeds in a sealed container with ~80% humidity for several days at 37 °C before assessing germination efficiency. While nearly 100% of both WT and *hira-1* mutant seeds germinated before the ageing treatment, seeds of both genotypes failed to germinate after 10 days of storage under high temperature and humidity showing the efficiency of this treatment (Figure 6b). At the intermediate time points after four, six and seven days of treatment, significantly fewer *hira-1* mutant seeds germinated compared to WT, underscoring the reduced seed vigor caused by the absence of HIRA. 

Taken together, chromatin of mature embryos adopts a chromatin state depending on the H3.3 histone incorporation pathway and is characterized by a very low H3.1/H3.3 ratio. In the absence of the HIR complex, the predominant H3.3 incorporation machinery, the H3 histone content in seeds is reduced and the nuclease accessibility of seed chromatin increased, a molecular phenotype accompanied by the alteration of two important seed traits—seed dormancy and vigor. 

## 3. Discussion

### 3.1. Embryos in Dry Seeds Have a Particular Chromatin Organization 

Enrichment in histone H3 variants varies through the cell cycle, with higher levels of H3.1 in S and G2 stages of the cell cycle. Furthermore, a global loss of H3.1 and enrichment in H3.3 was observed when cells completed their last cell cycle before differentiation or switching to the endocycle program [48,55]. In the Arabidopsis dry seed, most cells of the embryo are arrested in G1 [56,57]. Only during imbibition, the proportion of 4C nuclei increases in the embryo cell population, resulting from the onset of DNA replication and coinciding with radicle extrusion and therefore completion of the germination process [57]. In agreement with these studies, we observed very low *HTR1* and *HTR9* transcript levels in mature siliques and dry seeds and detected *de novo* accumulation of these transcripts during imbibition. In addition, none of the atypical H3 genes [16,46] are expressed in the seed (Figure S2e). In contrast to H3.1, transcripts encoding the histone H3.3 or HIRA proteins remain high in the dry seed. As a result, chromatin in the embryo shows a low H3.1/H3.3 ratio. This is not the only peculiarity of the histone variant repertoire in dry seed embryos, as recent reports revealed enrichment of seed chromatin in a particular H2B variant, termed H2B.8 [58,59]. It is interesting to speculate that these different features of seed chromatin contribute to the highly condensed chromatin state and the small nuclei that characterize the dry seed [60]. These specific features may potentially confer desiccation tolerance associated with this quiescent state in orthodox seeds that can survive drying, such as Arabidopsis. 

Due to the difficulty of performing chromatin immunoprecipitation on seed chromatin, little information is available concerning the genomic distribution of histone PTMs at the dry seed stage [61]. In leaf tissue, H3.3 is mainly enriched at the 3’ end of transcriptionally active genes [30,31,32,45] and carries PTMs associated with active transcription [62]. It remains to be explored whether in seeds H3.3 is enriched also in hetero- chromatin or if H3.1 may be retained at certain genomic sites and whether high H3.3 enrichment has repercussions on the PTM landscape. New histone deposition during imbibition and seedling establishment [57,63] may present a window of opportunity to reprogram chromatin structure by incorporating new sets of variants and to set different PTMs [9] to switch from the seed to the seedling transcriptome [49,64]. 

### 3.2. Role of HIRA in Controlling Nucleosome Occupancy and Transcription 

In line with the observation that H3 supply in the dry seed mostly depends on the H3.3 deposition pathway, we find reduced H3 content and altered chromatin accessibi- lity in *hira-1* mutant seeds. Deficient histone recycling and *de novo* deposition during transcription [44] taking place during seed maturation likely contribute to this phenotype. Indeed, during transcription, chromatin organization is continuously challenged as nucleosomes are displaced and disrupted and histones evicted to allow passage of the RNA polymerase. In mammals, the HIR complex ensures *de novo* deposition and histone recycling [44,65]. While not all molecular details have been dissected in plants, the composition of the HIR complex is conserved in Arabidopsis [42,43]. The molecular phenotype in *hira-1* mutant seedlings includes reduced nucleosomal occupancy [43] coherently with our observations in dry seeds. It can be expected that ATRX that is expressed in dry seeds (Figure S2f) contributes to H3.3 deposition [32], but whether *atrx* mutants also show seed dormancy and germination defects has not been explored yet. Besides HIRA, recycling of H3 histones during transcription is also ensured by the histone chaperones FACT and SPT6 [28]. Mutants in SSRP1, one of the two FACT subunits in Arabidopsis, show reduced seed dormancy [66]. Therefore, even though both FACT and HIRA are involved in histone recycling during transcription and ensure nucleosomal occupancy [43,44,67], their loss has opposite effects on seed dormancy, suggesting that they may differentially impact distinct regions of the genome.

The altered chromatin organization in *hira-1* mutants might affect gene expression by facilitating binding of transcription factors through exposure of binding sites, by easing RNA pol II processing through the chromatin template or by causing altered co-transcriptional events such as alternative splicing, which is widespread during germination [64] and influenced by the elongation rate [68]. In addition, reduced nucleosomal occupancy may also negatively affect chromatin state switches from active to repressive chromatin at seed developmental genes. 

### 3.3. Altered Chromatin Organization in hira-1 Links to Changes in Seed Dormancy

Seed dormancy is an important trait preventing pre-harvest sprouting and allowing the plant to adjust germination of its seeds to seasonal changes. To achieve this, both intrinsic signals and environmental stimuli need to be integrated to properly time germination. Besides plant hormones [69] and redox signaling [70], gene expression control at the level of chromatin has been shown to regulate dormancy. Many mutants affecting chromatin organization influence dormancy by modulating *DOG1* transcript levels. This is the case for *rdo2* (*tfIIs*) [26] and *hub1* mutants [22], which show lower *DOG1* transcript levels and reduced dormancy. While we did not observe any difference in transcript levels of genes involved in synthesis and catabolism of the plant hormones ABA, GA and in the ethylene signaling component EIN2 in *hira-1* mutant seeds, a moderate increase of *DOG1* mRNA was found in *hira-1* mutant seeds (Figure S6). Regulation of *DOG1* expression may therefore contribute to the increased dormancy phenotype observed in *hira-1* mutant seeds, but the exact mode of regulation remains a question for further investigation. Removing HIRA in the *rdo2 (tfIIs)* and *hub1* mutant backgrounds results in seeds that are more dormant than WT. It can therefore be speculated that reduced nucleosome occupancy caused by deficient histone recycling and deposition in the absence of HIRA might facilitate transcription of certain genes. This may in turn lessen the requirement for TFIIS and histone ubiquitination during transcription elongation. 

### 3.4. HIRA Contributes to Seed Vigor 

In addition to the dormancy phenotype in seeds of *hira-1* mutants, we also investigated whether altered chromatin organization could alter seed vigor. This complex trait was assessed by following the ability of mutant seeds to germinate in stressful saline conditions and by their ability to withstand accelerated ageing treatment. The seed vigor of *hira-1* mutants is lower compared to WT seeds, which suggests that DNA accessibility can control this trait, with regards to the role of HIRA-mediated histone deposition in transcriptional regulation [42,71,72]. Seed germination does not seem to strictly depend on *de novo* transcription but rather on translation of stored mRNAs [73]. It has nevertheless been proposed that the synthesis of new transcripts during imbibition is required for germination vigor [74] and for adjusting the germination program when seeds face unfavorable stressful conditions, a hypothesis that is in agreement with our observations. In addition, higher sensitivity of *hira-1* seeds to accelerated ageing may be a consequence of increased DNA damage. Indeed, cellular damage to macromolecules including DNA occurs during seed storage, for example, through reactive oxygen species [70,75], and is repaired in imbibed seeds [76,77]. Due to the failure to properly organize DNA in nucleosomal structures in combination with low cellular repair activities in the dry seed, *hira-1* mutant seeds may accumulate DNA damage to an extent that renders germination impossible. In line with this hypothesis, HIRA has been shown to play a role in chromatin reassembly after DNA double strand breaks [78] and in transcription recovery after exposure of mammalian cells to UV stress [71]. 

Seed dormancy and vigor play central roles in the adjustment of plant populations to their environment; however, the molecular mechanisms that integrate environmental signals to define germination timing remain poorly understood. Our work sets another piece to this complex puzzle, revealing a role for the histone chaperone HIRA in controlling histone flow, and consequently, chromatin organization in dry seeds and its contribution to seed vigor and germination under stressful conditions. Further research will show whether HIRA affects the expression of specific genes, alters global transcription efficiency or influences transcript resetting after DNA repair during imbibition. Based on these observations, it will be exciting to explore how enrichment in histone variants and their post-translational modifications is modulated in seed embryos in the field, where seeds face various adverse conditions and undergo multiple rounds of dormancy cycling. 

## 4. Materials and Methods

### 4.1. Plant Material

All *Arabidopsis thaliana* mutant lines are in the Columbia background. T-DNA insertions were obtained from Nottingham Arabidopsis Stock Center (NASC): *hira-1* (WiscDsLox362H05), *hub1-5* (SALK_044415C) and *rdo2-2 / tfIIs-2* (SALK_027259). The *hira-1* mutant line complemented by a construct expressing HIRA-GFP under control of its endogenous promoter was a courtesy of F. Berger [42]. Double mutant plants *hira-1 hub1-5* and *hira-1 rdo2-2* were obtained by genetic crosses. The transgenic lines expressing HTR1-GFP [50] and HTR5-GFP [46] under control of their endogenous promoters were kindly provided by Y. Fang and F. Berger, respectively.

To release physiological dormancy, seeds were stratified at 4 °C for one to four days before sowing, and plants were grown at 20 °C under long days (16 h of light / 8 h of dark). Seeds for germination tests were harvested dormant. To maintain dormancy, seeds were stored at harvest at −20 °C.

### 4.2. Germination Assays

Dormancy was alleviated by stratification at 4 °C for different times and germination assays performed at 10 °C or 25 °C in darkness in three biological replicates of at least 50 seeds for each genotype in 9-cm petri dishes on a layer of cotton wool covered by a filter paper sheet soaked with water or with water containing ABA (10^−6^ M), GA_3_ (10^−3^ M) or ethylene (50 ppm). Germination was recorded daily, and seeds were scored as germinated once the radicle protruded through the seed coat. 

For salinity stress, seeds were sterilized in 70% ethanol / 0.05% SDS, washed in 95% ethanol and sawn on plates with 1x MS medium supplemented with 50 or 100 mM NaCl. For controlled seed deterioration treatment, seeds were incubated at 37 °C in a sealed plastic container with saturated KCl solution at the bottom (resulting in air humidity of about 80% at 37 °C) for 4, 6, 7 or 10 days, respectively, before being sawn on filter paper. Seeds were stratified for two or three days at 4 °C and germinated at 20 °C under long days (16 h of light / 8 h of dark).

### 4.3. Confocal Imaging

Dry seed embryos from plants expressing HTR1-GFP [50] or HTR5-GFP [46] and cotyledons of two-days-old plantlets from the same seed batch were mounted in 1× PBS. Dry seeds were manually dissected from seeds imbibed for 1 h in water. Confocal imaging was performed using a Zeiss Axioimager microscope (Zeiss, Oberkochen, Germany) equipped with a high-speed Yokogawa CSUX1 spinning disk confocal, an ORCA-flash 4.0 digital camera (Hamamatsu, Hamamatsu, Japan) and a 40X water objective. GFP was excited at 488 nm, and the emission was collected between 500 and 560 nm. 

### 4.4. RNA Extraction

Total RNA was extracted from 20mg of seeds using a modified CTAB method as described by [79]. Alternatively, seeds were ground in liquid nitrogen and taken up in solubilization buffer (100 mM Tris-HCl pH 9,5; 150 mM NaCl, 5 mM DTT; 1% Sarkozyl) followed by phenol-chloroform extraction and precipitation in isopropanol. After precipitation and two washes in 70% ethanol, RNA was resuspended in water and purified a second time using RNAzol (MRC) according to the manufacturer’s instructions. RNA from flowers and two-day-old seedlings were extracted using the RNAzol method only. The RNA concentration and purity were checked using a Nanodrop (Neo Biotech, Nanterre, France). 

### 4.5. RT-qPCR 

A total of 2 µg of total RNA was reverse transcribed with oligo(dT)15 using M-MLVreverse transcriptase (Promega, Charbonnières, France). cDNAs were diluted 1:3 and used in quantitative PCR with the LightCycler 480 SYBR Green I Master kit on the Roche LightCycler 480. Transcript levels of interest were normalized to *MON1* (*At2g28390*) [80] or to *CB5-E* (*At5g53560*), *RHIP1* (*At4g26410*) and *TIP41* (*At4g34270*) [81] using the comparative threshold cycle method. Primers used can be found in Table S1. 

### 4.6. Western Blot

Proteins were extracted from about 30 mg of dry seeds in solubilization buffer containing 250 mM Tris-HCl pH 7.5, β-mercaptoethanol 0.07% and protease inhibitors (Roche), precipitated with TCA (10% *v*/*v*) and washed three times in 80% acetone before being taken up in 2× Laemmli buffer. Histone H3 was detected with an anti-H3 antibody (dilution 1:3000, ab1791, Abcam, Paris, France,). Immunoblot chemiluminescence was revealed using ECL protein gel blotting detection reagents (Clarity Western ECL Substrate, Bio-Rad, Marnes-la-Coquette, France). Densitometric analysis of immunoreactive protein bands was performed on non-saturated signals using Image Lab software (Bio-Rad, France) and normalized to actin using monoclonal anti-actin antibody (dilution 1:3000, clone 10-B3, A0480, Sigma-Aldrich, Saint Quentin-Fallavier, France).

### 4.7. MNase Digestion

A total of 400 mg of dry seeds were ground to a fine powder in liquid nitrogen and resuspended in extraction buffer (0.25 M sucrose, 10 mM Tris-HCl pH8, 10 mM MgCl_2_, 1 mM Triton X-100, 5 mM β-mercaptoethanol and proteinase inhibitors (Roche Diagnostics, Meylan, France). Filtered through miracloth and then through a 40 µM nylon filter, nuclei were collected by centrifugation at 1400× *g* and then resuspended in MNase buffer (10 mM Tris-HCl pH 7.5, 15 mM NaCl, 15 mM KCl, 1 mM CaCl_2_ supplemented with proteinase inhibitors). Nuclei extractions were adjusted to 200 ng/µL with MNase buffer after determining DNA concentration with a Nanodrop. Thirty units of MNase (Takara Bio Europe, Saint-Germain-en-Laye, France) were added to a Master tube, containing 300 µL of each sample, and 50 µL collected at 0, 1, 4, 16 and 64 min in 2× Stop solution (50 mM EDTA, 50 mM EGTA and 1% SDS supplemented with proteinase K). After incubation for 30 min at 37 °C, DNA was extracted with CTAB buffer (2% CTAB, 100 mM Tris-HCl pH8, 1.5M NaCl, 20 mM EDTA pH8) and chloroform before precipitation with isopropanol. DNA was taken up in TE buffer (10 mM Tris-HCl pH 7.5, 5 mM EDTA pH8) and treated by RNAse A / T1 (Thermo Scientific, Villebon sur Yvette, France) for 30 min at 37 °C. The DNA concentration was determined using a Nanodrop and equal amounts for each sample were run on a 1.5% agarose gel. 

## 5. Conclusions

In summary, our study sheds light on the chromatin organization in dry seeds, enriched in the replacement histone variant H3.3. We identify a role for the histone chaperone HIRA in controlling histone H3 levels and chromatin accessibility in the dry seed. Our results further show reduced seed vigor and enhanced seed dormancy in *hira-1* mutants, revealing HIRA as a positive regulator of seed germination. Besides the well-documented role of transcriptional and post-transcriptional mechanisms in this developmental step, our findings therefore highlight the role of epigenetic components in the regulation of the major traits involved in germination phenology. These results open avenues to decipher the contribution of chromatin organization in orchestrating changes in the highly regulated gene expression patterns required for developmental transitions. 

## Data Availability

The data presented in this study are available on request from the corresponding author.

## Supplementary Materials

The following are available online at https://www.mdpi.com/article/10.3390/ijms22084031/s1, Figure S1: *HTR2, HTR3, HTR13* and *HTR4* expression at different developmental stages, Figure S2: Dynamic expression of H3 histone chaperones during seed imbibition and germination, Figure S3: H3.1 and H3.3 expression in *hira-1* mutant seeds, Figure S4: Gene expression in *hira-1* mutant seeds, Figure S5: Growth and dormancy phenotypes of single and multiple *hira-1, hub1-5* and *rdo2-2* mutants, Figure S6: *DOG1* expression in *hira-1* mutant seeds, Table S1: Primers used in this study.
