# Supplementary material for: The Histone Chaperone HIRA Is a Positive Regulator of Seed Germination
Source: Int J Mol Sci. 2021 Apr 14;22(8):4031. doi: 10.3390/ijms22084031 (PMC8070706; doi:10.3390/ijms22084031)
Supplement: Supplementary file 1 [file ijms-22-04031-s001.pdf]

Supplementary Figures and Legends

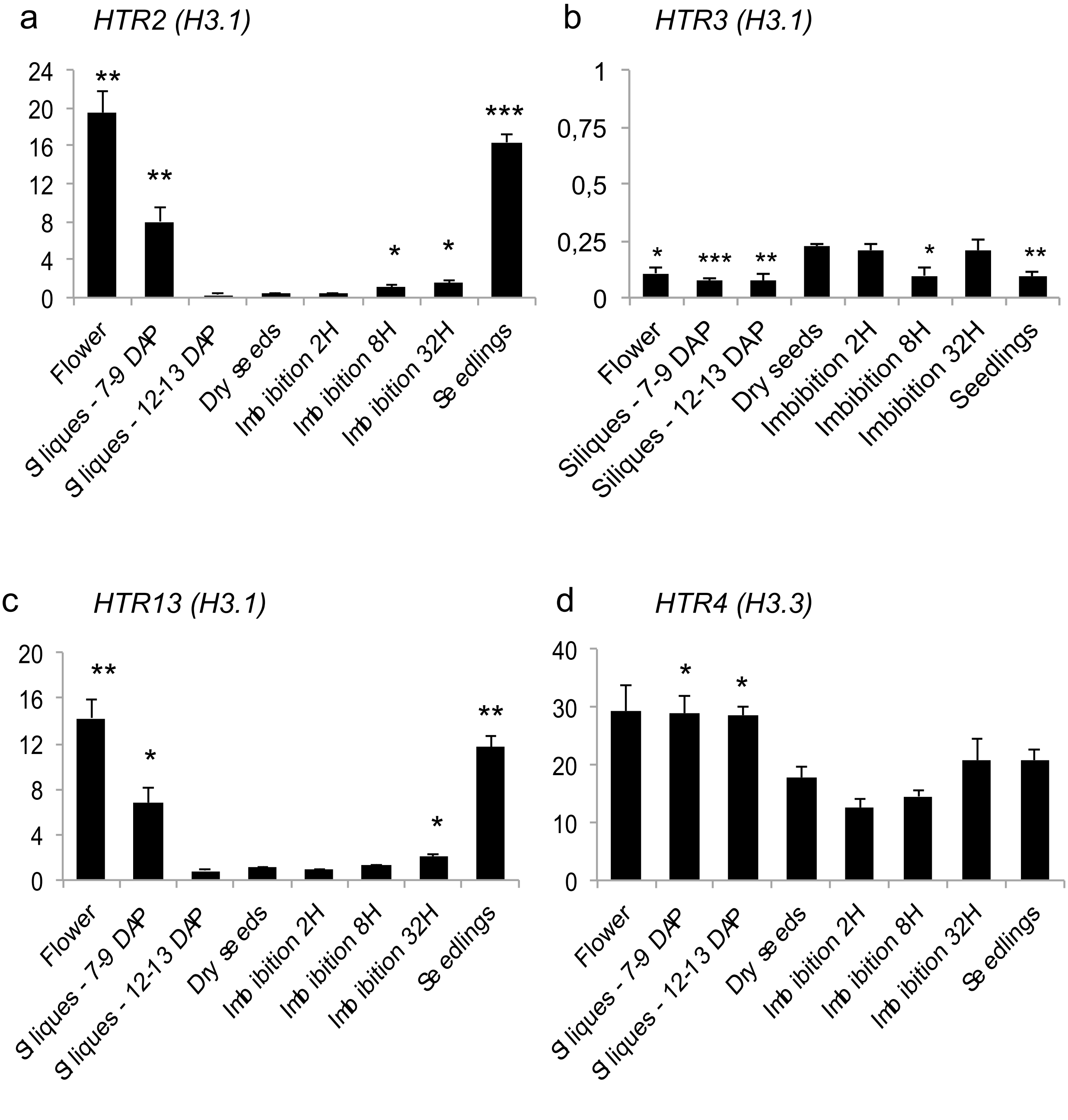

Figure S1. *HTR2, HTR3, HTR13* and *HTR4* expression at different developmental stages

Relative transcript levels of three genes encoding H3.1 (*HTR2* (**a**), *HTR3* (**b**) and *HTR13* (**c**)), as well as one gene encoding H3.3 (*HTR4 (**d**)*) in flowers, siliques (7-9 and 12-13 days after pollination (DAP)), dry seeds and seeds imbibed for 2 hours (H), 8H or 32H as well as 2 days old seedlings. Transcript levels are normalized to *MON1* (*At2g28390*). Error bars correspond to the SEM of three biological replicates and statistical significant differences relative to dry seeds were determined using a two-sided Student t-test (* $p< 0,05$; ** $p < 0,01$; *** $p < 0,001$).

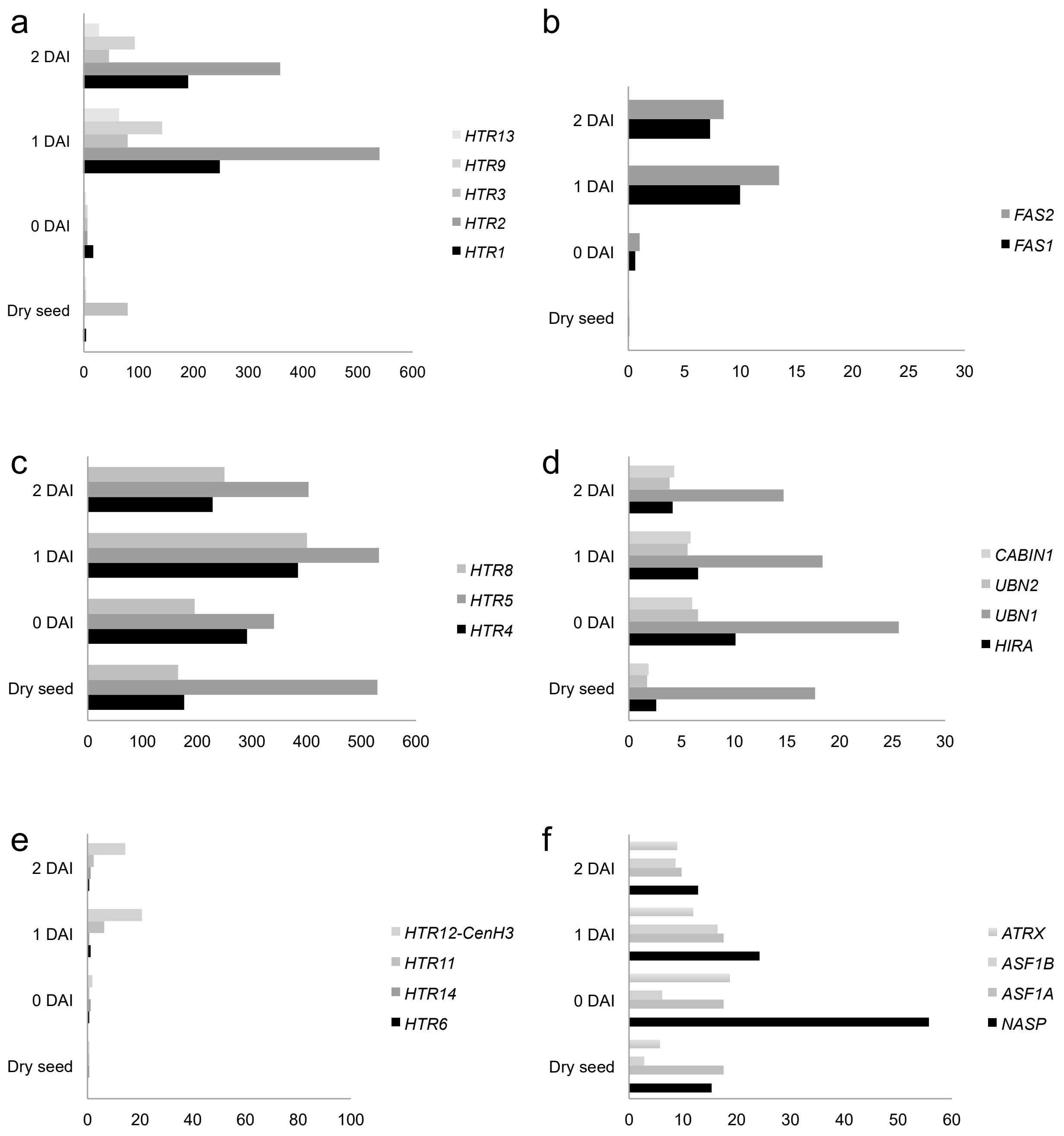

Figure S2: Dynamic expression of H3 histone chaperones during seed imbibition and germination

Expression in FPKM (Fragments Per Kilobase Million) of genes encoding H3 histones: replicative variants (**a**) *HTR1, HTR2, HTR3, HTR9* and *HTR13*; replacement variants (**c**) *HTR4, HTR5* and *HTR8*, the atypical variants H3.6 (*HTR6*), H3.14 (*HTR14*), H3.11 (*HTR11*) and the centromeric variant CenH3 (*HTR12*) (**e**) as well as genes encoding known H3 histone chaperones; including the CAF-1 complex (**b**), the HIRA complex (**d**) and genes encoding ATRX, NASP and ASF1a and b (**f**) in dry seeds, seeds after 4 days of stratification (0 Days after imbibition (DAI)) and after 1 or 2 days under long day conditions at 22°C (1 DAI, 2 DAI). Expression data are from [1]. *HTR10* (*H3.10*) and *H3.15 (HTR15)* are not expressed in these tissues.

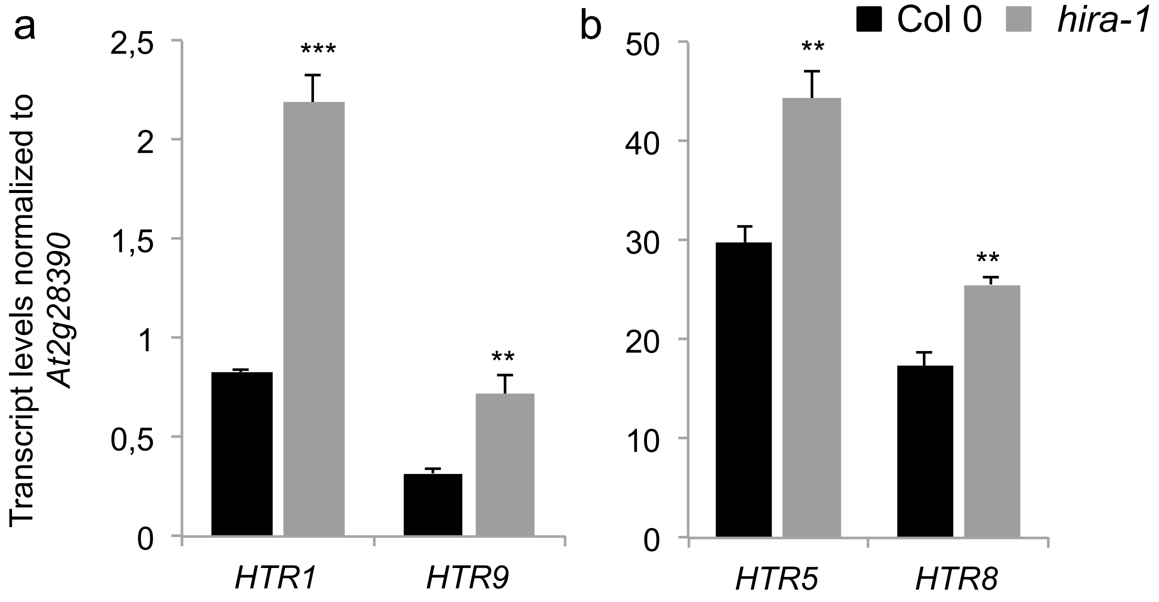

Figure S3: H3.1 and H3.3 expression in *hira-1* mutant seeds

Relative transcript levels of two genes encoding H3.1 (**a**, *HTR1, HTR9*) or H3.3 (**b**, *HTR5, HTR8*) in WT and *hira-1* mutant dry seeds. Error bars correspond to the SEM of four biological replicates and statistical significant differences relative to WT were determined using a two-sided Student t-test (** p< 0,01; *** p < 0,001).

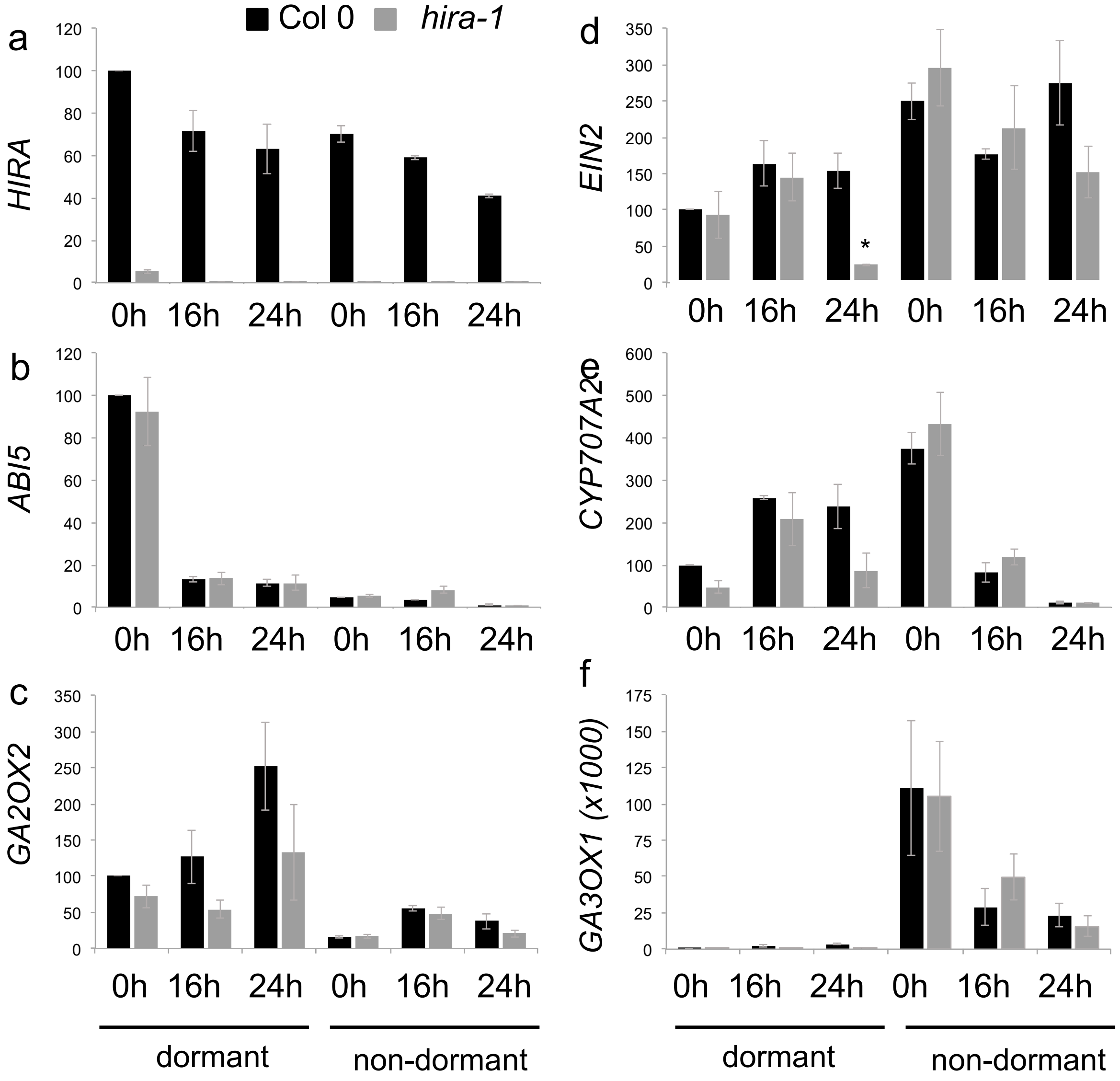

Figure S4: Gene expression in *hira-1* mutant seeds

Relative transcript levels of *HIRA* (**a**), *ABA INSENSITIVE 5 (ABI5)* (**b**), *GIBBERELLIN 2-OXIDASE 2 (GA2OX2)* (**c**), *ETHYLENE-INSENSITIVE PROTEIN2 (EIN2)* (**d**), *CYTOCHROME P450, FAMILY 707, SUBFAMILY A (CYP707A)* (**e**) and *GIBBERELLIN 3-OXIDASE 1 (GA3OX1)* (**f**) in dormant or non-dormant seeds (after 48 hours of imbibition) with or without incubation for 16 or 24 hours at 25°C in darkness. Error bars indicate SD for three biological replicates.

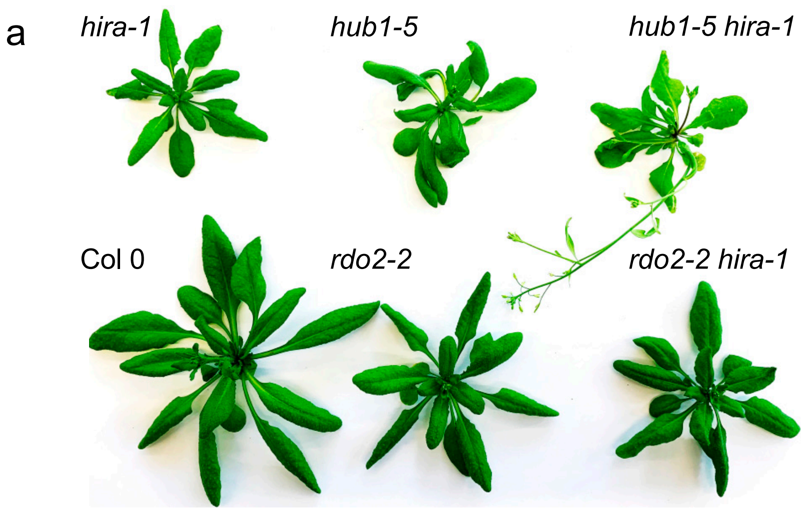

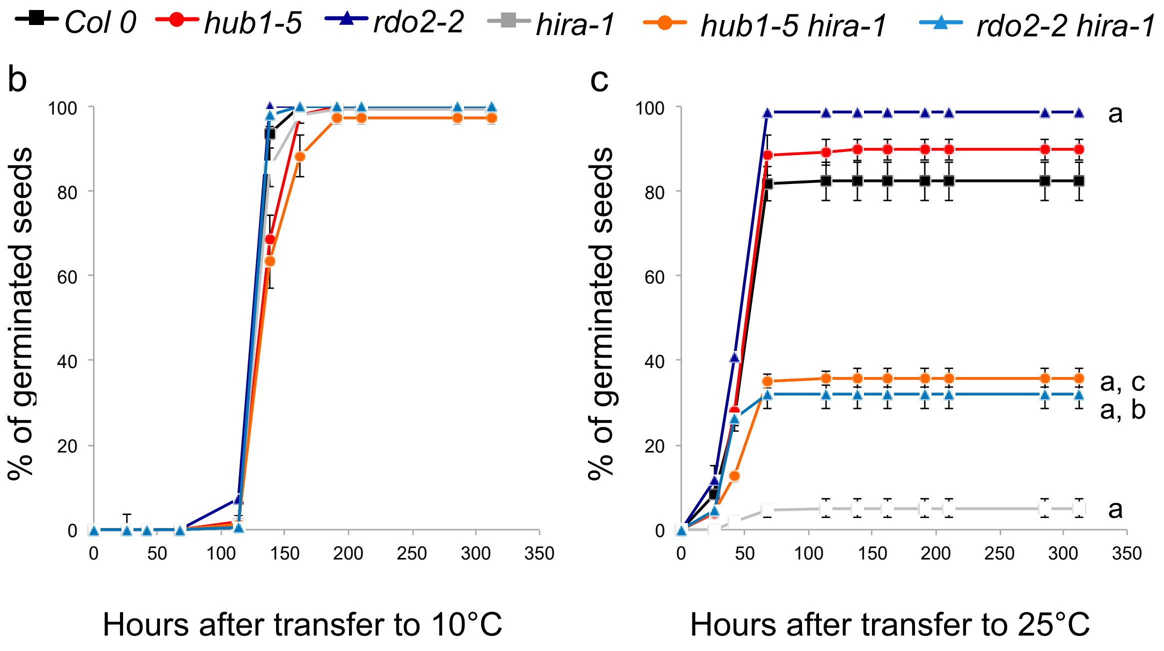

Figure S5: Growth and dormancy phenotypes of single and multiple *hira-1, hub1-5* and *rdo2-2* mutants

(**a**) Representative images of WT, *hira-1, hub1-5, rdo2-2, rdo2-2 hira-1* and *hub1-5 hira-1* mutant plants. (**b**-**c**) Same as Figure 5; data obtained with an independent seed batch. Germination of freshly harvested seeds of Col 0 (black), *hub1-5* (red), *rdo2-2* (dark blue), *hira-1* (grey), *hub1-5 hira-1* (orange) and *rdo2-2 hira-1* (light blue) at 10°C (seed viability test; a) or at 25°C (dormancy test; b) in darkness. Means of biological triplicates with SEM are shown. Statistical significant differences were determined using a two-sided Student t-test (a = p < 0,01 relative to Col 0; b = p < 0,01 relative to *hub1-5*; c = p < 0,01 relative to *rdo2-2*).

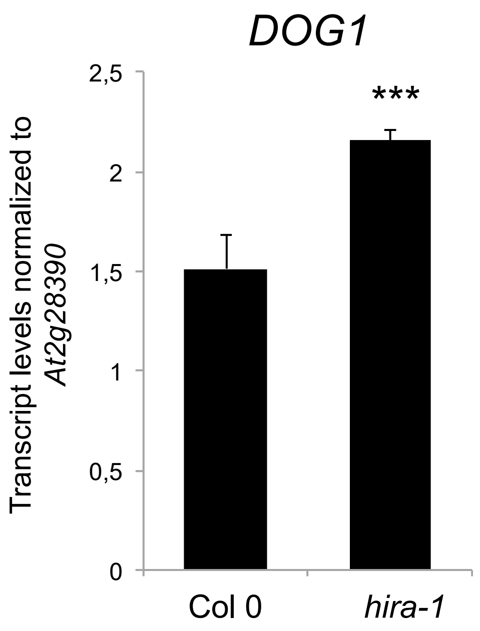

Figure S6: *DOG1* expression in *hira-1* mutant seeds

Relative *DOG1* transcript levels in WT and *hira-1* mutant dormant dry seeds. Error bars correspond to the SEM of four biological replicates and statistical significant differences relative to WT were determined using a two-sided Student t-test (*** p < 0,001).

Table S1. Primers used in this study.

Gene	Forward and Reverse primers (5' to 3')
MON1 (At2g28390)	AACTCTATGCAGCATTTGATCCACT<br>TGATTGCATATCTTTATCGCCATC
HTR1 (At5g65360)	AGCGATCTCACGAACCAAAC<br>GAAATCCACCGGAGGAAAAG
HTR2 (At1g09200)	GCAGACGGCTAGGAAATCAA<br>TTCCGGGTCTGAATCTGTGT
HTR3 (At3g27360)	CTGAAGTGTCTTCCAGATGT<br>ACAAGTCACTAAGACCTGAA
HTR4 (At4g40030)	AATAAAGATGGCTCGTACCA<br>CTTACGGATTTCACGGAGAG
HTR5 (At4g40040)	TTGCACTACGTGAAATTCGT<br>CACAAGGTATGCTTCAGCAG
HTR8 (At5g10980)	AAGAGCTAACACCGCGTGAC<br>AGGAAATCAGCACCAACCAC
HTR9 (At5g10400)	TAATCTCTGTGCGATTCATGCT<br>CGAAAACGAAAAGAGACAGCTT
HTR13 (At5g10390)	AAGCACCAAGAAAGCAACTC<br>CTTACGGATCAGAAGCTCAG
CB5-E (At5g53560)	TGAAGAAGTTTCAAAGCACAACA<br>TCATCCATGAATGGAGTCACA
TIP41 (At4g34270)	GTGAAAACTGTTGGAGAGAAGCAA<br>TCAACTGGATACCCTTTCGCA
RHIP1 (At4g26410)	GAGCTGAAGTGGCTTCCATGAC<br>GGTCCGACATACCCATGATCC
HIRA (At3g44530)	AACAAGACCAGAACTCAAGA<br>CTTTAACAACGCCTAACTGAG
DOG1 (At5G45830)	TCTCGTCTCGACAAGTCAGC<br>ACCAAATCTGCCAAGTCCAT
ABI5 (At2g36270)	AACATGCATTGGCGGAGTTG<br>GCAATTTCGGTTGTGCCCTT
GA2OX2 (At1g30040)	ATGGTGAAGGTGGGGTTTGG<br>CTCCCGTTAGTCATAACCTGAAG
GA3OX1 (At1g15550)	CATCACCTCAACTACTGCGATA<br>TTAGCTGGAGAGCAGCTTGG
CYP707A2 (At2g29090)	CTCGCCAAAACAAGTACGGG<br>AGCCGCCTCTGGACTACTTTA
EIN2 (At5g03280)	CACCACTCTGCTTGTATCGTC<br>TCGTCTTCGTTCCTCCTTGT

References

1. Kawakatsu, T.; Nery, J.R.; Castanon, R.; Ecker, J.R. Dynamic DNA methylation reconfiguration during seed development and germination. *Genome Biol.* **2017**, *18*, 171, doi:10.1186/s13059-017-1251-x.
